# Cognitive and Kidney Function: Results from a British Birth Cohort Reaching Retirement Age

**DOI:** 10.1371/journal.pone.0086743

**Published:** 2014-01-22

**Authors:** Richard J. Silverwood, Marcus Richards, Mary Pierce, Rebecca Hardy, Naveed Sattar, Charles Ferro, Caroline Savage, Diana Kuh, Dorothea Nitsch, On behalf of the NSHD scientific and data collection teams

**Affiliations:** 1 Faculty of Epidemiology and Population Health, London School of Hygiene and Tropical Medicine, London, United Kingdom; 2 MRC Unit for Lifelong Health and Ageing, University College London, London, United Kingdom; 3 BHF Glasgow Cardiovascular Research Centre, University of Glasgow, Glasgow, United Kingdom; 4 Department of Renal Medicine, Queen Elizabeth Hospital, Birmingham, United Kingdom; 5 School of Immunity and Infection, College of Medical and Dental Sciences, University of Birmingham, Birmingham, United Kingdom; University Of São Paulo, Brazil

## Abstract

**Background:**

Previous studies have found associations between cognitive function and chronic kidney disease. We aimed to explore possible explanations for this association in the Medical Research Council National Survey of Health and Development, a prospective birth cohort representative of the general British population.

**Methods:**

Cognitive function at age 60–64 years was quantified using five measures (verbal memory, letter search speed and accuracy, simple and choice reaction times) and glomerular filtration rate (eGFR) at the same age was estimated using cystatin C. The cross-sectional association between cognitive function and eGFR was adjusted for background confounding factors (socioeconomic position, educational attainment), prior cognition, and potential explanations for any remaining association (smoking, diabetes, hypertension, inflammation, obesity).

**Results:**

Data on all the analysis variables were available for 1306–1320 study members (depending on cognitive measure). Verbal memory and simple and choice reaction times were strongly associated with eGFR. For example, the lowest quartile of verbal memory corresponded to a 4.1 (95% confidence interval 2.0, 6.2) ml/min/1.73 m^2^ lower eGFR relative to the highest quartile. Some of this association was explained by confounding due to socioeconomic factors, but very little of it by prior cognition. Smoking, diabetes, hypertension, inflammation and obesity explained some but not all of the remaining association.

**Conclusions:**

These analyses support the notion of a shared pathophysiology of impaired cognitive and kidney function at older age, which precedes clinical disease. The implications of these findings for clinical care and research are important and under-recognised, though further confirmatory studies are required.

## Introduction

The brain and kidneys may be considered end organs on parallel trajectories, subject to shared cardiovascular risk factors [1]. Chronic kidney disease (CKD) is associated with underlying factors such as inflammation, homocysteine, oxidative stress, anaemia, hypertension, and diabetes [1] which are also important markers for cerebrovascular disease which leads to impaired cognition. Correspondingly, impaired estimated glomerular filtration rate (eGFR) [2], [3] and albuminuria [4] have been found to be associated with cognitive decline and dementia in population-based studies, with evidence that this is the case for vascular dementia in particular [5]. Although most studies to date have considered how the level or rate of change of cognitive function differs according to levels of kidney function, and some have taken this to indicate a direction of causality from kidney function to cognitive impairment [2], the exact basis for the relationship is remains uncertain and a pathophysiological process affecting both brain and kidney remains a viable explanation [3].

Whilst it is fairly well understood that CKD is associated with the cardiometabolic syndrome phenotype, which in turn is prospectively associated with further cognitive decline [6], [7], it is unclear whether similar associations can be found for pre-clinical kidney function impairment. If there was an association between subtle cognitive impairment and kidney function preceding clinical CKD, then this would suggest a shared pathophysiology which operates before the onset of clinical disease. Indeed, one study found that a cross-sectional association between impaired eGFR and cognition in old age was explained by childhood IQ [8]. The association between childhood IQ and old age cognition was mediated by adult socioeconomic position (SEP) and vascular risk factors (blood pressure, HbA1c and cholesterol) [8], suggesting that poor cognitive development lies along a path leading to a risk environment for chronic physical diseases.

Kidney function is often assessed using eGFR calculated from measured creatinine using one of several equations developed over recent years [9], [10]. However, at higher levels of eGFR, as seen in general population samples, cystatin C has been found to be better correlated than creatinine with true GFR [11]. Cystatin C has also been found to be a stronger predictor of the risk of death and cardiovascular events than creatinine [12].

The Medical Research Council National Survey of Health and Development (NSHD) provides a unique opportunity to explore the pathways by which kidney and cognitive function may be associated. This study is a prospective birth cohort representative of the general British-born population with data at the age of 60–64 years allowing us to assess kidney function using serum cystatin C-based eGFR and cognitive function via a battery of four tests (five measures in total), and to explore possible explanations for this association using data collected throughout life. We hypothesised that kidney function and cognitive function were cross-sectionally associated at age 60–64, due in part to confounding by socioeconomic factors but also due to mechanisms linked to smoking, diabetes, hypertension, inflammation, and obesity.

## Materials and Methods

### Ethics Statement

The study protocol received ethical approval from the Central Manchester Research Ethics Committee for data collection taking place in Manchester, Birmingham, Cardiff and London. Ethical permission was given by the Scotland A Research Ethics Committee for the data collection taking place in Edinburgh. Written informed consent was obtained from the study member at each stage of data collection.

### Data Availability

Bona fide researchers can apply to access the NSHD data via a standard application procedure (further details available at: http://www.nshd.mrc.ac.uk/data.aspx).

### Participants

The NSHD is a socially stratified sample of 5362 singleton children born within marriage in one week in March 1946 in England, Scotland and Wales, who have been followed up 23 times since birth [13]. Between October 2006 and February 2011 (at 60–64 years), 2856 eligible study members (those known to be alive and with a known address in England, Scotland or Wales) were invited for an assessment at one of six Clinical Research Facilities (CRFs). Invitations were not sent to those who had died (n = 778), were living abroad (n = 570), had previously withdrawn from the study (n = 594) or had been lost to follow-up (n = 564). If study members were unable or unwilling to come to one of the CRFs they were offered a slightly less comprehensive examination carried out in their own home by a trained nurse. Of those invited, 2229 (78.0%) were assessed: 1690 (59.2%) attended a clinic and 539 (18.9%) had a home visit [14]. All participants underwent informed consent, and the study adhered to the principles outlined in the Helsinki declaration.

### Measures

We utilised five measures of cognitive function derived from four different tests, all of which were carried out at the clinic or home visit at age 60–64.

#### Verbal memory

This was a classic test of ‘episodic’ memory; a 15-item word list learning task where the study member was shown each word for 2 seconds then was asked to write down as many of these from memory as possible, in any order, within 1 minute. This was repeated twice for a total of three learning trials. Each trial was scored for the total number of different correct words recalled and the scores across the three learning trials were summed.

#### Letter search

This was a ‘cancellation’ task, testing mental speed, visual scanning, and focused concentration. It required the study member to cross out as many targets – in this case the letters P and W – embedded in a 30×20 letter matrix, as quickly and accurately as possible within 1 minute. The test required study members to begin at the top of the page and work from left to right, line by line, without going backwards at any point. We derived two different measures from this test: speed (the last target crossed out by the time limit] and accuracy (the number of targets correctly crossed out as a percentage of the letters which should have been crossed out up to that point).

#### Simple reaction time

This was a standard manual reaction time task which required study members to press one button as quickly as possible following a visual signal using a custom-built device. Eight practice trials were given, followed by 20 real trials (each consisting of a single signal). To avoid anticipation there was a variable delay (1–3 seconds) between each response and the next signal. The mean reaction time across the 20 real trials was calculated.

#### Choice reaction time

Using the same device as above, this manual reaction time task required study members to press one of four buttons as quickly as possible corresponding to which of the numbers 1 to 4 appeared in the signal screen. Eight practice trials were given, followed by 40 real trials (each consisting of a single signal). The same variable delay was used as for simple reaction time. The mean reaction time for correct responses across the 40 real trials was calculated.

At the clinic or home visit at age 60–64 years, blood samples were taken and processed according to a standardised protocol. Cystatin C was measured by an automated particle-enhanced immunoturbidimetric assay at the Glasgow Royal Infirmary, Department of Clinical Biochemistry with an inter-assay variability of <3%. eGFR was calculated from measured cystatin C using the formula of Inker et al [11].

### Potential Confounders

**Figure 1:**
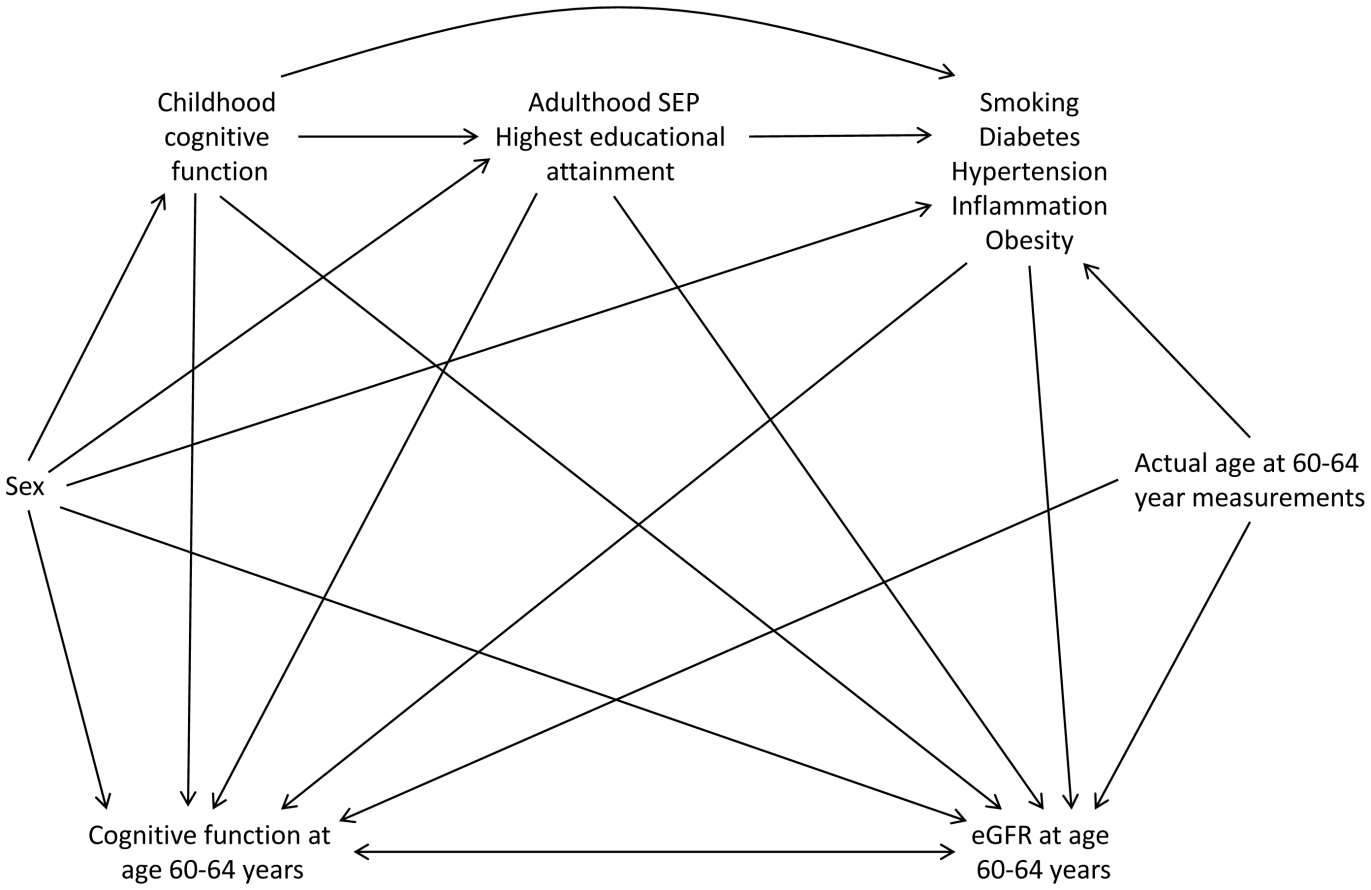
Causal diagram showing inter-relation of variables. Arrows show the assumed direction of causal influence, with the double-headed arrow between cognitive function at age 60–64 years and eGFR at age 60–64 years indicating that we make no assumption about the direction of causality between these two variables. SEP, socio-economic position; eGFR, estimated glomerular filtration rate.

Using prior knowledge and results from the existing literature we constructed a causal diagram containing variables known or hypothesised to be associated with cognitive and kidney function to aid in the selection of potential confounders (Figure 1). We considered sex, age, adulthood SEP, highest educational qualifications, childhood cognitive function, smoking, diabetes, hypertension, inflammation, and obesity to all be potential common causes of cognitive function and eGFR at age 60–64. In order to block all the open backdoor paths between cognitive function and eGFR at age 60–64 it is necessary to adjust for all of these variables [15]. Here we consider adulthood SEP and highest educational qualification to be background confounders whose confounding effect we first wish to remove. Our main interest is in the extent to which the other potential confounders (childhood cognitive function, smoking, diabetes, hypertension, inflammation, and obesity) explain any remaining association.

SEP at age 53 was defined as the highest occupational class derived from study member’s and their spouse’s reported occupations based on the British Registrar General’s Social Classification [16]: ‘I and II professional and managerial’, ‘III non-manual’, ‘III manual skilled’, or ‘IV and V manual semi or unskilled’. Highest educational attainment was ascertained when the study member was age 26 and categorised as ‘no qualifications’, ‘vocational only’, ‘ordinary (‘O’ level or equivalent)’, ‘advanced (‘A’ level or equivalent)’, or ‘higher (degree or equivalent)’.

Childhood cognitive function was tested at age 8 in a school setting using four different tests: a 50-item vocabulary test, a 35-item verbal comprehension (sentence completion) test, mechanical reading (pronunciation) using the same words as for vocabulary, and a 60-item non-verbal ability (picture intelligence) test. Each test was scored for the total number of correct responses and the scores across the four tests were summed.

Lifetime smoking (between age 20 and 53) was classified as ‘never smoker’, ‘predominantly non-smoker’, ‘predominantly smoker’, or ‘lifelong smoker’ [17].

Diabetes (self-reported doctor diagnosed diabetes by age 60–64, being on diabetes treatment at age 60–64, HbA1c level at age 60–64) and hypertension (previously derived systolic blood pressure (SBP) latent trajectory between ages 36 and 53 [18], being on anti-hypertensive treatment at age 60–64, measured SBP at age 60–64) were considered. HbA1c was analysed using a Tosoh G7 analyser at Addenbrooke’s Hospital in Cambridge. Two measures of SBP were taken with an OMROM 705 with the participant seated, with the minimum of the two readings used in this analysis. The remaining diabetes and hypertension data were derived from questionnaire responses.

C-reactive protein (CRP) at age 60–64 was measured by a particle-enhanced immunoturbidimetric assay using a Siemens Dimension Xpand analyser at the MRC Human Nutrition Research laboratory in Cambridge with an average inter-assay variability over this period of 3.3%.

Adult heights and weights were measured at ages 36, 43, 53 and 60–64 years, and self-reported at ages 20 and 26. Body mass index (BMI) was calculated as weight in kilograms divided by height in metres squared and overweight at each age defined using the standard cut point of 25 kg/m^2^. The age at which a study member first became overweight (26, 36, 43, 53, 60–64 or never) was then derived assuming that once an individual became overweight they remained overweight [19].

### Statistical Analyses

Due to the cross-sectional nature of the eGFR and cognitive function data, and our lack of prior belief in either direction of causality over the other, it would be reasonable to consider either eGFR or cognitive function as the outcome in our models. We used eGFR as the distribution was approximately normal, which was not true of several of the cognitive function measures.

The cross-sectional associations between eGFR and each of the five measures of cognitive function at age 60–64 were first examined using linear regression. As there was evidence of non-linearity in some of the associations we used quartiles of the cognitive function measures as exposures. Models were adjusted for sex and age at cystatin C measurement.

The associations between eGFR and i) childhood cognitive function; ii) SEP at age 53; iii) highest educational attainment; iv) lifetime smoking; v) diabetes; vi) hypertension; vii) current CRP; viii) current BMI and ix) age at first overweight were then examined using linear regression, with adjustment for sex and age at cystatin C measurement.

In the analysis we aimed to first remove the confounding effects of adulthood SEP and highest educational qualification from out estimate of the association between eGFR and cognitive function at age 60–64. We then explored the extent to which the remaining potential confounders (childhood cognitive function, smoking, diabetes, hypertension, inflammation, and obesity) accounted for any remaining association. Sequentially adjusted linear regression models for eGFR on each of the five measures of cognitive function at age 60–64 were fitted on the subsample of subjects with complete data on all explanatory variables included in the analysis. Model 1 was minimally adjusted for sex and age at cystatin C measurement; Model 2 was additionally adjusted for SEP at age 53 and highest educational qualification; Model 3 was additionally adjusted for childhood cognitive function; Model 4 was additionally adjusted for lifetime smoking, diabetes, hypertension, current CRP, current BMI and age at first overweight.

In all models sex differences were investigated by adding interaction terms and using the likelihood ratio test (LRT). LRTs for linear trend were applied where appropriate, and non-linear associations were examined by adding quadratic terms to the models and using the LRT.

The analysis was performed using Stata 12 [20].

## Results

Of the 2229 study members who were assessed at age 60–64, 2036 had data on cystatin C and one or more of the five measures of cognitive function so were included in the initial analyses. At age 60–64 median eGFR in the study, weighted according to the original social class-stratified sampling, was 96.8 ml/min/1.73 m^2^. The median HbA1c was 5.7% (38.8 mmol/mol), median SBP was 133 mmHg, median BMI was 27.5 kg/m^2^, and median CRP was 2.2 mg/L. Further characteristics of the sample are shown in Table 1.

**Table 1:** Distributions of variables.

	Males		Females		Total	
Variable	N	Median (IQR)^A^	N	Median (IQR)^A^	N	Median (IQR)^A^
Cognitive function at age 60–64 years						
Verbal memory	955	23 (8)	1028	25 (8)	1983	24 (8)
Letter search – speed	966	239 (79)	1038	239 (85)	2004	239 (79)
Letter search – accuracy	966	92.0 (10.3)	1038	92.0 (10.3)	2004	92.0 (10.3)
Simple reaction time	960	269 (63)	1029	271 (63)	1989	270 (63)
Choice reaction time	958	614 (110)	1026	622 (106)	1984	618 (108)
Cystatin C-based eGFR at age 60–64 years (ml/min/1.73 m^2^)	983	99.7 (19.4)	1053	95.6 (16.7)	2036	96.8 (18.7)
Cognitive function at age 8	870	87 (39)	939	90 (39)	1809	88 (38)
HbA1c at age 60–64 years (%)^B^	942	5.7 (0.5)	1013	5.7 (0.5)	1955	5.7 (0.5)
Systolic blood pressure at age 60–64 years (mmHg)	981	136 (22)	1042	130 (22)	2023	133 (21)
Body mass index at age 60–64 years (kg/m^2^)	975	27.8 (5.1)	1046	27.0 (6.8)	2021	27.5 (6.1)
C-reactive protein (mg/L)	980	2.1 (2.3)	1049	2.2 (2.8)	2029	2.2 (2.6)
	**N**	**n (%** A **)**	**N**	**n (%** A **)**	**N**	**n (%** A **)**
Socioeconomic position at age 53 years	912		1001		1913	
I and II professional and managerial		582 (59.1)		599 (55.2)		1181 (57.1)
III non-manual		176 (21.8)		240 (25.8)		416 (23.9)
III manual skilled		114 (14.6)		88 (10.4)		202 (12.4)
IV and V manual semi or unskilled		40 (4.5)		74 (8.6)		114 (6.7)
Highest educational attainment	927		1000		1927	
No qualifications		288 (37.6)		302 (37.1)		590 (37.3)
Vocational only		51 (6.0)		95 (11.3)		146 (8.8)
Ordinary ('O' level or equivalent)		136 (14.0)		268 (26.6)		404 (20.6)
Advanced ('A' level or equivalent)		291 (30.1)		276 (20.9)		567 (25.3)
Higher (degree or equivalent)		161 (12.3)		59 (4.1)		220 (8.0)
Lifetime smoking	922		1000		1922	
Never smoker		249 (25.0)		334 (32.5)		583 (28.9)
Predominantly non-smoker		362 (38.4)		347 (32.5)		709 (35.3)
Predominantly smoker		201 (22.8)		191 (19.7)		392 (21.2)
Lifelong smoker		110 (13.8)		128 (15.3)		238 (14.6)
Self-reported diabetes by age 60–64 years	892		971		1863	
No		827 (92.0)		922 (94.8)		1749 (93.5)
Yes		65 (8.0)		49 (5.2)		114 (6.5)
On diabetes treatment at age 60–64 years	983		1053		2036	
No		926 (94.0)		1018 (96.6)		1944 (95.4)
Yes		57 (6.0)		35 (3.4)		92 (4.6)
Midlife systolic blood pressure trajectory^C^	974		1044		2018	
Normal		924 (94.6)		974 (92.0)		1898 (93.3)
Increaser/high		50 (5.4)		70 (8.0)		120 (6.7)
On anti-hypertensive treatment at age 60–64 years	983		1053		2036	
No		687 (68.8)		775 (70.8)		1462 (69.9)
Yes		296 (31.2)		278 (29.2)		574 (30.1)
Age at first overweight (years)	888		991		1879	
26		197 (24.8)		127 (13.9)		324 (19.0)
36		173 (21.3)		103 (11.9)		276 (16.2)
43		126 (15.2)		136 (14.1)		262 (14.6)
53		147 (15.3)		229 (22.9)		376 (19.3)
60–64		62 (6.5)		108 (11.7)		170 (9.3)
Never		183 (16.9)		288 (25.6)		471 (21.5)

Restricted to study members non-missing for at least one measure of cognitive function at age 60–64 years and cystatin C-based estimated glomerular rate (eGFR) at age 60–64 years.

IQR, inter-quartile range.

A Weighted according to the original social class-stratified sampling.

B HbA1c (mmol/mol) = (HbA1c (%) –2.15)×10.929.

C Latent trajectories previously derived from systolic blood pressure data at ages 36, 43 and 53 years [18].

There was strong evidence that verbal memory and choice reaction time were linearly associated with eGFR at age 60–64 (both *P*<0.001; Table 2), with lower cognitive function associated with lower eGFR (i.e. worse kidney function). The lowest quartile of verbal memory corresponded to a 4.7 (95% confidence interval (CI) 3.0, 6.4) ml/min/1.73 m^2^ lower eGFR relative to the highest quartile. There was strong evidence (*P*<0.001) of an association with simple reaction time, but this did not appear to be linear, with the fastest quartile of reaction time instead corresponding to markedly higher eGFR than the remaining quartiles. There was somewhat weaker evidence of an association with letter search speed, but no evidence of an association with letter search accuracy.

**Table 2:** Linear regression models for cystatin C-based estimated glomerular rate (eGFR) at age 60–64 years (ml/min/1.73 m^2^, dependent variable) by cognitive function quartile (independent variable).

Cognitive function quartile	n (%) in thisgroup	Mean (SD) eGFR(ml/min/1.73 m^2^)	Coeff	95% CI	*P* overall^A^	*P* for trendA,B
Verbal memory (n = 1983)					<0.001	<0.001
1 (highest)	508 (25.6)	96.4 (12.8)	(ref)			
2	453 (22.8)	94.4 (14.1)	−2.1	−3.9, −0.4		
3	488 (24.6)	94.2 (14.4)	−2.6	−4.3, −0.9		
4 (lowest)	534 (26.9)	92.3 (15.4)	−4.7	−6.4, −3.0		
Letter search – speed (n = 2004)					0.17	0.03
1 (fastest)	432 (21.6)	95.1 (14.2)	(ref)			
2	519 (25.9)	94.3 (13.7)	−0.8	−2.6, 1.0		
3	548 (27.3)	94.4 (14.3)	−0.9	−2.6, 0.9		
4 (slowest)	505 (25.2)	93.5 (15.5)	−2.0	−3.8, −0.2		
Letter search – accuracy (n = 2004)					0.49	0.74
1 (highest)	519 (25.9)	94.6 (14.3)	(ref)			
2	538 (26.8)	93.9 (14.5)	−0.8	−2.5, 0.9		
3	469 (23.4)	94.9 (14.2)	0.4	−1.4, 2.1		
4 (lowest)	478 (23.9)	93.9 (14.8)	−0.8	−2.5, 1.0		
Simple reaction time (n = 1989)					<0.001	
1 (fastest)	503 (25.3)	96.5 (12.6)	(ref)			
2	512 (25.7)	93.3 (15.0)	−3.5	−5.2, −1.8		
3	487 (24.5)	94.4 (13.7)	−2.0	−3.8, −0.3		
4 (slowest)	487 (24.5)	93.3 (15.8)	−3.2	−4.9, −1.5		
Choice reaction time (n = 1984)					0.002	<0.001
1 (fastest)	508 (25.6)	96.4 (13.0)	(ref)			
2	493 (24.9)	95.0 (14.0)	−1.3	−3.0, 0.5		
3	506 (25.5)	92.9 (14.7)	−3.1	−4.8, −1.4		
4 (slowest)	477 (24.0)	93.2 (15.5)	−2.7	−4.4, −0.9		

A Likelihood ratio test.

B Only reported where appropriate.

All models adjusted for sex and age at cystatin C measurement.

Higher childhood cognitive function was also associated with higher eGFR at age 60–64 (*P*<0.001; Table 3). There was strong evidence that SEP at age 53, highest educational attainment, lifetime smoking and age at first overweight were linearly associated with eGFR (all *P*<0.001), with lower (i.e. manual) SEP, lower qualifications, greater lifelong exposure to smoking and becoming overweight earlier corresponding to lower eGFR. Self-reported diabetes, being on diabetes treatment, increaser/high midlife SBP trajectory and being on anti-hypertensive treatment were associated with lower eGFR (all *P*≤0.02). There was evidence of non-linear (quadratic) trends for both HbA1c and CRP, with minimum eGFR corresponding to a HbA1c of 7.9% and a CRP of 60 mg/L. Consequently quadratic terms for HbA1c and CRP were added to Models 4 and 5 below. Higher current BMI was associated with lower eGFR (*P*<0.001). There was some evidence of a sex difference in the association with current BMI, with females with higher BMI having particularly low eGFR. For simplicity, combined male and female results are presented in Table 3, but the appropriate sex interaction term was added to Models 4 and 5 below.

**Table 3:** Linear regression models for cystatin C-based estimated glomerular rate (eGFR) at age 60–64 years (ml/min/1.73 m^2^, dependent variable).

Explanatory variable	n	Coeff	95% CI	*P* overall^A^	*P* for trendA,B
Childhood cognitive function (per 30 units^C^)	1823	1.3	0.6, 2.0	<0.001	
Socioeconomic position at age 53 years	1923			<0.001	<0.001
I and II professional and managerial		(ref)			
III non-manual		−2.4	−3.9, −0.8		
III manual skilled		−3.1	−5.1, −1.0		
IV and V manual semi or unskilled		−3.1	−5.8, −0.5		
Highest educational attainment	1942			<0.001	<0.001
No qualifications		(ref)			
Vocational only		1.8	−0.8, 4.3		
Ordinary ('O' level or equivalent)		3.4	1.6, 5.2		
Advanced ('A' level or equivalent)		3.1	1.5, 4.7		
Higher (degree or equivalent)		4.9	2.8, 7.1		
Lifetime smoking	1936			0.003	<0.001
Never smoker		(ref)			
Predominantly non-smoker		−0.7	−2.2, 0.8		
Predominantly smoker		−1.5	−3.3, 0.3		
Lifelong smoker		−3.9	−6.0, −1.8		
Self−reported diabetes by age 60–64 years	1877	−3.1	−5.7, −0.5	0.02	
On diabetes treatment at age 60–64 years	2052	−3.0	−5.9, −0.1	0.04	
HbA1c at age 60–64 years (per %)	1970			<0.001	
Linear		−10.4	−15.5, −5.2		
Quadratic		0.7	0.3, 1.0		
Midlife systolic blood pressure trajectory (Increaser/high vs normal)^D^	2033	−3.3	−5.9, −0.7	0.01	
On anti−hypertensive treatment at age 60–64 years	2052	−3.7	−5.0, −2.4	<0.001	
Current systolic blood pressure (per 20 mmHg^C^)	2033	0.4	−0.3, 1.1	0.21	
Current C−reactive protein (per 10 mg/L^C^)	2045			<0.001	
Linear		−3.7	−5.3, −2.1		
Quadratic		0.3	0.1, 0.5		
Current body mass index (per 5 kg/m^2^ C)	2030	−2.4	−3.0, −1.7	<0.001	
Age at first overweight (years)	1887			<0.001	<0.001
26		−3.8	−5.8, −1.8		
36		−4.4	−6.5, −2.3		
43		−3.3	−5.4, −1.2		
53		−0.5	−2.4, 1.4		
60–64		−1.5	−4.0, 0.9		
Never		(ref)			

A Likelihood ratio test.

B Only reported where appropriate.

C Approximately 1 standard deviation.

D Latent trajectories previously derived from systolic blood pressure data at ages 36, 43 and 53 years [18].

All models adjusted for sex and age at cystatin C measurement.

In subjects with complete data (n = 1306 to 1320; Table 4, Model 1) the minimally adjusted associations between cognitive function and eGFR were similar to those using all available data (n = 1983 to 2004; Table 2), though confidence intervals were somewhat wider due to the reduced sample size. These associations were all attenuated on adjustment for SEP at age 53 and highest educational attainment (Table 4, Model 2), though there remained some evidence of associations with verbal memory, simple reaction time and choice reaction time. Additional adjustment for childhood cognitive function (Model 3) had very little impact on the estimated associations. Additional adjustment for lifetime smoking, diabetes, hypertension, current CRP, current BMI, and age at first overweight (Table 5, Model 4) attenuated the results further. In these fully adjusted models there was little evidence of overall associations between cognitive function and eGFR (*P*≤0.15 for all models), though some of the individual regression coefficients were still relatively large and negative, indicating that that the observed associations had not been fully adjusted away.

**Table 4:** Linear regression models for cystatin C-based estimated glomerular rate (eGFR) at age 60–64 years (ml/min/1.73 m^2^, dependent variable) by cognitive function quartile at age 60–64 years (independent variable).

				Model 1				Model 2			
Cognitive function quartile	n	n (%)	Mean (SD) eGFR(ml/min/1.73 m^2^)	Coeff	95% CI	*P*overall^A^	*P* fortrend^A^	Coeff	95% CI	*P*overall^A^	*P* fortrend^A^
Verbal memory	1313					<0.001	<0.001			0.21	0.10
1 (highest)		352 (26.8)	96.6	(ref)				(ref)			
2		313 (23.8)	94.6	−2.1	−4.2, 0.0			−1.5	−3.6, 0.6		
3		320 (24.4)	94.8	−2.1	−4.1, 0.0			−0.8	−2.9, 1.4		
4 (lowest)		328 (25.0)	92.9	−4.1	−6.2, −2.0			−2.3	−4.6, 0.0		
Letter search – speed	1320					0.50	0.13			0.91	0.50
1 (fastest)		294 (22.3)	95.5	(ref)				(ref)			
2		359 (27.2)	94.8	−0.8	−2.9, 1.4			−0.5	−2.6, 1.6		
3		359 (27.2)	94.5	−1.1	−3.2, 1.1			−0.5	−2.7, 1.6		
4 (slowest)		308 (23.3)	94.1	−1.7	−3.9, 0.5			−0.8	−3.1, 1.4		
Letter search – accuracy	1320					0.97	0.75			0.99	0.83
1 (highest)		350 (26.5)	95.0	(ref)				(ref)			
2		353 (26.7)	94.6	−0.5	−2.5, 1.6			−0.1	−2.2, 1.9		
3		314 (23.8)	94.6	−0.4	−2.6, 1.7			−0.1	−2.2, 2.0		
4 (lowest)		303 (23.0)	94.7	−0.4	−2.5, 1.8			0.3	−1.9, 2.4		
Simple reaction time	1308					0.003	0.002			0.03	0.02
1 (fastest)		328 (25.1)	97.3	(ref)				(ref)			
2		334 (25.5)	94.2	−3.1	−5.2, −1.0			−2.7	−4.8, −0.6		
3		337 (25.8)	94.7	−2.4	−4.5, −0.4			−2.0	−4.1, 0.1		
4 (slowest)		309 (23.6)	93.4	−3.8	−5.9, −1.7			−2.9	−5.1, −0.8		
Choice reaction time	1306					0.02	0.004			0.11	0.06
1 (fastest)		347 (26.6)	96.7	(ref)				(ref)			
2		319 (24.4)	95.8	−1.1	−3.1, 1.0			−0.8	−2.9, 1.2		
3		340 (26.0)	93.4	−2.9	−5.0, −0.9			−2.5	−4.5, −0.4		
4 (slowest)		300 (23.0)	93.7	−2.6	−4.7, −0.5			−1.5	−3.7, 0.7		

A Likelihood ratio test.

Model 1: Adjusted for sex and age at cystatin C measurement.

Model 2: Additionally adjusted for socioeconomic position at age 53 years and highest educational attainment.

**Table 5:** Linear regression models for cystatin C-based estimated glomerular rate (eGFR) at age 60–64 years (ml/min/1.73 m^2^, dependent variable) by cognitive function quartile at age 60–64 years (independent variable).

	Model 3				Model 4			
Cognitive function quartile	Coeff	95% CI	*P* overall^A^	*P* for trend^A^	Coeff	95% CI	*P* overall^A^	*P* for trend^A^
Verbal memory			0.27	0.13			0.26	0.23
1 (highest)	(ref)				(ref)			
2	−1.4	−3.6, 0.7			−1.3	−3.4, 0.8		
3	−0.7	−2.9, 1.5			−0.1	−2.2, 2.1		
4 (lowest)	−2.1	−4.5, 0.2			−1.7	−4.0, 0.5		
Letter search – speed			0.92	0.52			0.96	0.97
1 (fastest)	(ref)				(ref)			
2	−0.5	−2.6, 1.7			−0.3	−2.4, 1.8		
3	−0.5	−2.6, 1.7			0.2	−1.9, 2.3		
4 (slowest)	−0.8	−3.0, 1.5			−0.2	−2.4, 2.0		
Letter search – accuracy			0.98	0.77			0.84	0.97
1 (highest)	(ref)				(ref)			
2	−0.1	−2.1, 2.0			0.4	−1.7, 2.4		
3	−0.1	−2.2, 2.0			−0.5	−2.5, 1.6		
4 (lowest)	0.3	−1.8, 2.5			0.4	−1.8, 2.5		
Simple reaction time			0.03	0.02			0.15	0.16
1 (fastest)	(ref)				(ref)			
2	−2.7	−4.8, −0.6			−2.3	−4.3, −0.2		
3	−2.0	−4.1, 0.1			−1.4	−3.4, 0.7		
4 (slowest)	−2.9	−5.0, −0.7			−1.9	−4.0, 0.3		
Choice reaction time			0.13	0.08			0.23	0.15
1 (fastest)	(ref)				(ref)			
2	−0.8	−2.9, 1.3			−0.9	−2.9, 1.2		
3	−2.4	−4.5, −0.4			−2.1	−4.1, −0.1		
4 (slowest)	−1.4	−3.6, 0.8			−1.1	−3.3, 1.1		

A Likelihood ratio test.

Model 3: Additionally adjusted for childhood cognitive function.

Model 4: Additionally adjusted for lifetime smoking, self-reported diabetes by age 60–64 years, being on diabetes treatment at age 60–64 years, HbA1c at age 60–64 years, midlife systolic blood pressure trajectory, being on anti-hypertensive treatment at age 60–64 years, systolic blood pressure at age 60–64 years, C-reactive protein at age 60–64 years, body mass index at age 60–64 years and age at first overweight.

## Discussion

In this large, population-based, prospective study we found cognitive function at age 60–64 years to be strongly associated with eGFR at the same age. Some of this association was explained by confounding due to socioeconomic factors, but very little of it was explained by prior cognitive function. The potential preceding adverse health behaviours of smoking and obesity, the clinical problems of diabetes and hypertension, and a biomarker for inflammation explained some but not all of the remaining association. In fully adjusted models weak evidence of an association between eGFR and reaction time (both simple and choice) remained.

Our findings are consistent with the emerging literature that poor kidney function, in particular progressive kidney disease, is associated with cognitive decline and dementia in the community [21], [5], [22], [3]. However, evidence of residual cross-sectional associations after adjusting for childhood cognition does not fully answer the question of association directionality, since kidney function had not been previously measured in this cohort.

It should be noted that these residual cross-sectional associations were not found by Munang et al [8]. These authors found a modest cross sectional association between eGFR and cognitive function in men in the Lothian 1921 cohort which was fully attenuated by adjustment for childhood cognition. This may be, however, because cystatin C eGFR is a better marker of true kidney function in this type of population [11] than the MDRD eGFR used by Munang et al, the formula for which was derived using a nephrology clinic CKD population [9].

A recent magnetic resonance study suggests that the association between kidney and cognitive function may be related to changes in intracranial deep white matter lesions which are progressive in people with chronic kidney disease [23]. The pathways by which these white matter lesions may develop are unclear. One hypothesis is that kidney disease is a marker for small vessel disease which may be present both in kidneys and the brain [24]. This would perhaps explain the cross-sectional association found, but not why some evidence for this association remained after adjustment for relevant biomarkers and preceding health behaviours that are associated with small vessel disease in the general population. In children with existing kidney disease, cognitive impairment appears to be associated with the severity of hypertension [25]. We adjusted our analyses for current SBP, midlife SBP trajectory and history of hypertension, but this adjustment may not have captured the entire range of blood pressure variability throughout life, or indeed across the day. Recent studies suggest that it is not only the level of blood pressure, but also its short-term variability which is related to later cerebrovascular outcomes [26], [27]. It also remains possible that these associations have no causal sequence but are explained by a common-cause factor that has not been measured herein.

These analyses support the notion of a shared pathophysiology of impaired cognitive and kidney function at older age, which precedes clinical disease. Hence, subtle cognitive impairment with poorer verbal memory and decreased reaction times may be present in some nephrology patients. Our analyses highlight some pathways by which socioeconomic inequalities manifest. Awareness of these associations and pathways may enhance the usefulness of clinic consultations. Pathways that connect kidney and cognitive function via social inequality may differ by setting, but shouldn’t be ignored, in particular because people with CKD have a substantive and complex pill burden. At the more severe end of CKD the pill burden undergoes frequent adaptations. Patients are expected to make fairly complex changes in their medication regimen on a frequent basis. To date it is unclear how much poor patient compliance may be due to misunderstandings caused by impaired cognitive function in the patient which is not allowed for by the clinical team. Communicating with a cognitively impaired patient requires frequent checks of patient understanding and is helped by asking the patient/carer to bring the prescribed drugs to the clinic consultation. Our findings also have important implications for research. For example, any large-scale study using questionnaires in people with CKD should spend some time validating these questionnaires to check whether the questions are understood by patients from all backgrounds in the way the researcher wants them to be, otherwise there will be a subtle measurement bias in the study. An important implication of this study and the existing body of work is that people with chronic kidney disease are potentially at higher risk for further decline of cognitive function [2], [21], [28]. Future studies will need to examine the role of kidney disease and blood pressure variability on progressive cognitive decline.

There are several strengths to this analysis. Data were prospectively collected using standardised protocols, and the availability of appropriate variables across the life course allowed us to remove the principal sources of background confounding, adjust for prior cognition, and explore potential explanations for the remaining association.

There were also limitations to the study. Although study members remaining in the NSHD at the time of data collection at age 60–64 years were broadly representative of native-born adults living in England, Scotland, and Wales [14], the samples for our sequentially adjusted analyses included only 59% of such subjects. Whilst our complete case analysis approach may possibly have led to the disproportionate exclusion of the more disadvantaged and those in poorest health, this would not necessarily have affected the pattern of results observed. We used cystatin C eGFR as a proxy for kidney function as gold-standard measurements, such as the clearance of exogenous filtration markers, were not available. We were unable to fully answer the question of association directionality as kidney function had not been previously measured in this cohort. We employed observational data and, despite being confident that we identified and included all the important confounders, bias due to unknown and/or unmeasured confounders cannot be ruled out. Finally, whilst we had good coverage of different British regions and social class groupings, as the NSHD study population is all white our findings cannot necessarily be extrapolated to the non-white British population.

In conclusion, cognitive and kidney function in late mid-life are strongly associated with one another, though some of this is explained by socioeconomic factors across the life course. Socioeconomic inequality manifests itself through a variety of pathways leading to chronic disease, including reduced kidney function and cognitive impairment.
